# Cutaneous and Developmental Effects of *CARD14* Overexpression in Zebrafish

**DOI:** 10.3390/biomedicines10123192

**Published:** 2022-12-08

**Authors:** Avital Baniel, Limor Ziv, Zohar Ben-Moshe, Ofer Sarig, Janan Mohamad, Alon Peled, Gideon Rechavi, Yoav Gothilf, Eli Sprecher

**Affiliations:** 1 Division of Dermatology, Tel Aviv Sourasky Medical Center, Tel Aviv 64239, Israel; 2 Cancer Research Center, Sheba Medical Center, Ramat Gan 52620, Israel; 3 Department of Neurobiology, The George S. Wise Faculty of Life Sciences, Tel-Aviv University, Tel Aviv 6997801, Israel; 4 Department of Human Molecular Genetics and Biochemistry, Sackler Faculty of Medicine, Tel-Aviv University, Tel Aviv 6997801, Israel

**Keywords:** zebrafish, *CARD14*, psoriasis

## Abstract

Background: Gain-of-function mutations in *CARD14* have recently been shown to be involved in the pathogenesis of psoriasis and pityriasis rubra pilaris (PRP). Those mutations were found to activate the NF-kB signaling pathway. Objective: Zebrafish is often used to model human diseases in general, and in skin disorders more particularly. In the present study, we aimed to examine the effect of *CARD14* overexpression in zebrafish with the aim to validate this model for future translational applications. Methods: We used light microscopy, scanning electron microscopy, histological analysis and whole mount in situ hybridization as well as real-time PCR to ascertain the effect of *CARD14* overexpression in the developing zebrafish. Results: Overexpression of human *CARD14* had a marked morphological and developmental effect on the embryos. Light microscopy demonstrated a characteristic cutaneous pattern including a granular surface and a spiky pigment pattern. In situ hybridization revealed keratinocytes of uneven size and shape. Scanning electron microscopy showed aberrant production of actin microridges and a rugged keratinocyte cell surface, reminiscent of the human hyperkeratotic phenotype. Developmentally, overexpression of *CARD14* had a variable effect on anterior-posterior axis symmetry. Similar to what has been observed in humans with psoriasis or PRP, NF-kB expression was higher in *CARD14*-overexpressing embryos compared to controls. Conclusions: Overexpression of *CARD14* results in a distinct cutaneous pattern accompanied by hyperactivation of the NF-kB pathway, suggesting that the zebrafish represents a useful system to model *CARD14*-associated papulosquamous diseases.

## 1. Introduction

Psoriasis and pityriasis rubra pilaris (PRP) are common papulosquamous diseases which bear some overlapping features but have been traditionally considered as distinct clinical and histopathological entities [1]. Many genetic factors have been implicated in the pathogenesis of psoriasis [2]. In PRP, familial disease is rarer, and is estimated to account for approximately 5% of the cases [3]. Of interest, the study of the genetic basis of both diseases has revealed that they may share a common etiology. Indeed, familial cases of either psoriasis or PRP have been shown to be caused by mutations in the same gene, *CARD14*, encoding the caspase recruitment domain-containing protein 14 [4,5]. In fact, *CARD14* maps to the PSORS2 psoriasis-associated locus [6,7]. *CARD14* mutations have been shown to cause a wide range of phenotypes, recently termed CARD14-associated papulosquamous eruption (CAPE) [8]. Most disease-causing mutations in *CARD14* affect the coiled-coil domain of the protein and are gain-of-function variants [9]. In contrast, loss-of-function mutations in *CARD14* have been shown to cause an atopic dermatitis-like phenotype [9,10,11,12].

CARD14 is a member of the CAspase Recruitment Domain (CARD) family of proteins which mediate signal transduction in apoptotic and inflammatory processes. These proteins share a CARD domain comprising 6 or 7 anti-parallel alpha-helices which enable highly specific homophilic interactions between proteins involved in signal transduction [13,14]. CARD14 functions by activating the NF-kB signaling pathway as well as the p38 and JNK MAP kinase pathways, by forming a complex with BCL10 and the paracaspase MALT1 [15].

Despite its evolutionary distance, the zebrafish model shares many biological features with humans [16]. A homolog of *CARD14* has been found in several teleost fishes including the zebrafish [17], but its physiological function is not yet explored. Phylogenetic analysis of the two species results in a bootstrap value of 91% and the CARD domain, essential for binding BCL10 and activation of NFkB is conserved [17]. Since psoriasis- and PRP-causing *CARD14* mutations have been shown to result in a gain-of-function [4,18] and increased expression of CARD14 in the skin [4,15], we reasoned that overexpression of *CARD14* in zebrafish may result in a phenotype relevant to human diseases which may subsequently be of help in ascertaining the effect of potential treatments on CARD14-induced cell signaling.

## 2. Materials and Methods

### 2.1. DNA and RNA Injection to Zebrafish Embryos

A male and a female fish were brought together into a laying chamber towards the evening of the day prior to the injection. On the morning of the injection day, at first light, the embryos were collected as a single cell (0–30 min after fertilization). The embryos were arrayed on a 4% agarose injection plate.

For injection of human *CARD14* DNA (*hcard14*), the embryos were injected with *CARD14*-expression constructs of wildtype or PRP-causing mutation (p.Glu138del) which were generated as previously described [18]. Control embryos consisted of either mock-injected embryos or embryos injected with an empty DNA plasmid (pcDNA3.1).

For injection of zebrafish *card14* mRNA (*zfcard14)*, the coding sequence was PCR-amplified from larval cDNA (F,TCGGATTTAGGGATTTCAGA ; R,TATCTGGGTGTCATGCCTCA) and cloned into pCS2+ vector based on NEBuilder^®^ Assembly tool (http://nebuilder.neb.com, accessed on 3 June 2019) using the NEBuilder^®^ HiFi DNA Assembly Cloning Kit (New England BioLabs, Ipswich, MA, USA). Control embryos consisted of either *gfp m*RNA or *card14 m*RNA containing a stop codon replacing the start codon. Introduction of the stop mutation was accomplished by the Q5^®^ Site-Directed Mutagenesis Kit (New England BioLabs). Primers for mutagenesis were designed utilizing the NEBaseChanger^®^ tool (http://nebasechanger.neb.com, accessed on 3 June 2019). Cloning and mutagenesis were validated by direct sequencing. DNA or RNA were loaded into an internal filament of a capillary that was stretched with a micro pipette puller. The capillary was then connected to the injection device (air pressure microinjector-PV830 Pneumatic PicoPump, Sarasota, FL, USA). Approximately 1 nL of DNA was injected into a fertilized egg at a single cell stage. All zebrafish procedures were approved by the Tel-Aviv University Animal Care Committee (04-18-051) and conducted in accordance with the National Council for Animal Experimentation, Ministry of Health, Israel.

### 2.2. Whole Mount In Situ Hybridization (WISH)

Larvae samples were fixed in 4% PFA overnight at 4 °C and subsequently dehydrated by serial dilutions of methanol (25%, 50%, 75%, 100%) in phosphate-buffered saline solution (PBST) supplemented with 0.1% Tween-20. Dehydrated larvae were maintained at −20°C. For WISH, larvae were rehydrated by a reversed gradient of methanol in PBST. Then, depigmentation was performed with 5% H_2_O_2_ in PBST. Permeabilization was achieved by 10 μg/mL of proteinase K in PBST. Then, larvae were fixed in 4% PFA for 30 min at room temperature. Fixed larvae were washed by PBST (3×) and incubated in a pre-hybridization buffer [1 mM EDTA, 5XSSC (0.75 M NaCl, 75 mM sodium citrate, pH 7.0.), 2% Roche blocking powder, 50% formamide, 0.1%, 1 mg/mL Torula yeast RNA, 0.1% CHAPS, DEPC-treated ddH_2_O, Triton-X, 50 mg/mL heparin) at 65 °C overnight. On the next day, riboprobes designed using Primer3 (https://primer3.ut.ee/, accessed on 1 July 2019) (Table S1) were added and incubation was pursued for additional 48 h, prior to washing out of unbound riboprobes by 2X SSC and 0.2X SSC (each solution 3 × 30 min).

### 2.3. Scanning Electron Microscopy (SEM)

The samples were fixed by Kranovsky fixative 4% PFA, 2% glutaraldehyde (GA) in 0.1 M Cacodilate (Caco) buffer. Subsequently, samples were stained with 1% OsO4 in Caco 0.1 M buffer, dehydrated in an ethanol gradient (50%, 70%, 96%, and 100%), and critical-point dried using a critical-point dryer BAL-TEC CPD 030 (Leica Bio-Systems, Wetzlar, Germany). The dried samples were then put on aluminum stabs covered with carbon tape and coated with a thin layer of gold/palladium alloy in Edwards sputter coater. Sample visualization was attained by secondary electron detector in a high-resolution Ultra 55 SEM (Zeiss, Jena, Germany).

### 2.4. Histology

Larvae samples were fixed in 4% PFA, dehydrated in 70% ethanol and embedded in paraffin. Cross sections (2 μm thick) were cut using a Shandon M1R rotary microtome (Marshall Scientific, Hampton, NH, USA) and stained by hematoxylin and eosin (H&E).

### 2.5. RNA Purification

Fifty-100 mg of tissue were homogenized with 1 mL of TRI Reagent^®^ (Sigma-Aldrich, St. Louis, MO, USA) at room temperature for 5 min. Then, 0.2 mL of chloroform was added for every 1 mL of TRI Reagent^®^. Tubes were shaken for 15 s, incubated at room temperature for 15 min and then centrifuged at 12,000× *g* for 15 min at 4 °C. The supernatant was separated, 0.5 mL of isopropanol was added per 1 mL of TRI Reagent^®^. Samples were then incubated at room temperature for 10 min and centrifuged at 12,000× *g* for 10 min at 4 °C. The fluid was discarded and 1 mL of ethanol 75% was supplemented to the pellet for every 1 mL used in the preparation of the sample and then centrifuged at 7500× *g* for 5 min at 4 °C.

### 2.6. Real Time PCR

For quantitative real-time PCR (qPCR) analysis, cDNA was synthesized from 1000 ng of total RNA by the qScript kit (Quanta Biosciences, Gaithersburg, MD, USA). cDNA PCR amplification was performed using the PerfeCTa SYBR Green FastMix (Quanta Biosciences, Gaithersburg, MD, USA) on a StepOnePlus system (Applied Biosystems, Waltham, MA, USA) with gene-specific intron-crossing pairs of oligonucleotides (designed using primer3) (Table S2). Cycling conditions were: 95 °C, 20 s and then 95 °C, 3 s; 60 °C, 30 s for 40 cycles. Samples were analyzed in triplicates. For each primer set, standard curves were obtained with successive cDNA dilutions. Results were normalized to *ppiab* (NM_199957) mRNA levels. For list of primers, see Supplementary Material.

## 3. Results

### 3.1. Effect of CARD14 Overexpression on Zebrafish Larvae Development

**Figure 1:**
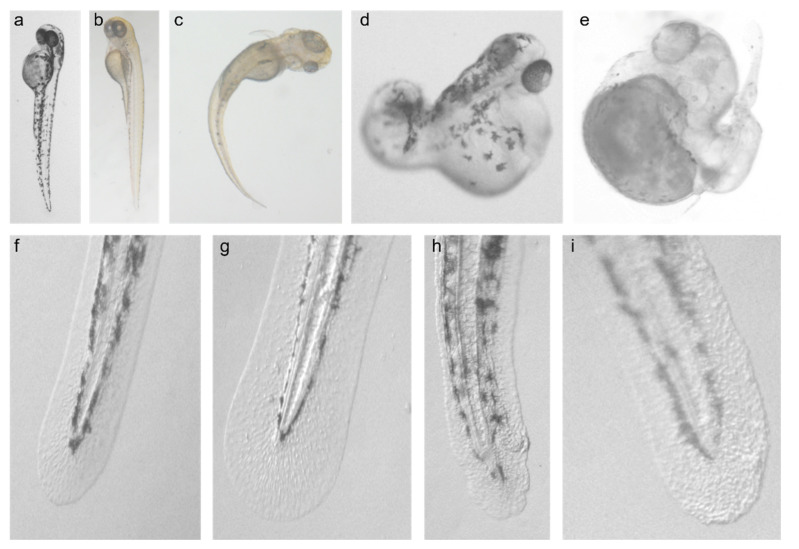
Developmental and cutaneous effects of *Card14* overexpression. Three days post-fertilization (dpf) uninjected (**a**) and *gfp*-RNA (**b**) injected larvae as compared to larvae injected with *zfCard14* RNA (**c**–**e**). The latter demonstrating a wide range of disturbed caudal development. Note asymmetric eye development in (**c**,**e**). Smooth surface, regular border and uniform pigmentation are seen in uninjected (**f**) and *gfp-*RNA injected (**g**) larvae; in contrast, irregular borders, spiky pigment and a “granular” surface are seen in larva injected with zf*card14* (**h**) and *hcard14* (**i**) RNA.

Zebrafish embryos were injected with RNA expressing zf*card14*, h*CARD14* and *gfp* as control. Overall, 197 larvae overexpressing either zfCARD14 (136) or hCARD14 (61) and 140 controls were examined. The larvae were examined from day 1 to 7 post fertilization. A markedly disturbed developmental pattern was observed, affecting mainly the caudal pole. Of note, the phenotype was abnormal albeit of variable severity with curved and irregularly shaped tails up to almost complete absence of tail development (Figure 1a–e). Cephalic development was normal at the exception of occasional asymmetrical eye development. Cardiac edema was also a common finding.

### 3.2. Effect of CARD14 Overexpression on Zebrafish Larvae Cutaneous Morphology

Meticulous examination of skin morphology revealed distinctive cutaneous morphological changes. The skin border of larvae injected with zf*card14* and h*CARD14* was irregular, showing a pattern of “hills and valleys”. In addition, larvae skin contained clusters of granules as opposed to the smoother and more uniform appearance of the surface of control larvae (Figure 1f–i). In contrast with control larvae in which symmetrically organized pigmented globules with uniform borders were seen, in larvae overexpressing CARD14, an asymmetrical spiky pattern of pigment was often observed (Figure 1h). As is the rule in zebrafish embryo injections, developmental abnormalities were occasionally noted in controls to some extent, but none showed a conspicuous and redundant developmental or cutaneous pattern as in CARD14 overexpressing larvae. Embryos injected with a vector carrying a PRP-causing mutation (p.Glu138del) showed the same cutaneous and developmental pattern.

### 3.3. Effect of CARD14 Overexpression on Keratinocyte Morphology

**Figure 2:**
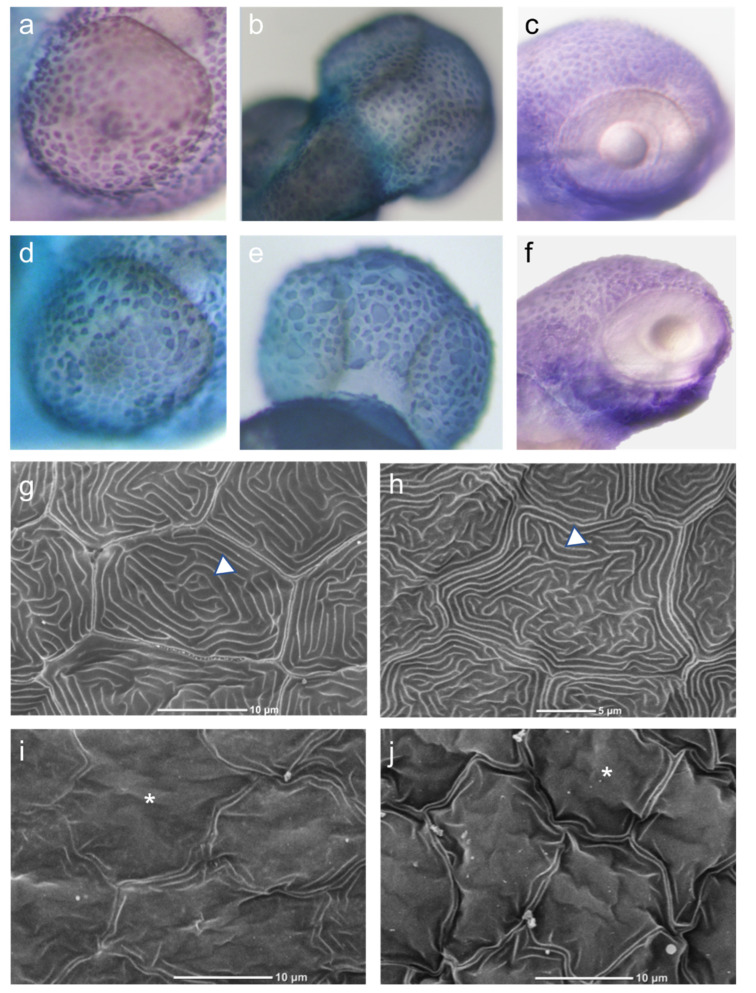
Keratinocyte morphology. Upper panel: Whole mount in situ hybridization with a probe recognizing cytokeratin1 mRNA (dark stain) at 2 dpf (**a**,**c,d,f**) and 3 dpf (**b**,**e**) demonstrating the distribution and morphology of keratinocytes over the eye (**a**,**d**) and over the head (**b**,**c,e**–**f**) areas. Control larvae were injected with an empty plasmid (**b**) or *gfp*RNA (**a,c**), and compared to larvae injected with h*CARD14* (**e**) or *zfcard14* (**d**,**f**). Note in both regions the uneven distribution and size as well as cell cluster formation as a consequence of *CARD14* overexpression as opposed to the even size and regular borders of control keratinocytes. Lower panel: Scanning electron microscopy of 2 dpf embryos. Uninjected (**g**) and *gfp-*RNA injected (**h**) embryos display a uniform and complete array of microridges (triangle), and smooth cell surface, while *zfcard14* (**i**) and *hCARD14* injected (**j**) embryos display uneven cell surface topography (asterisk).

**Figure 3:**
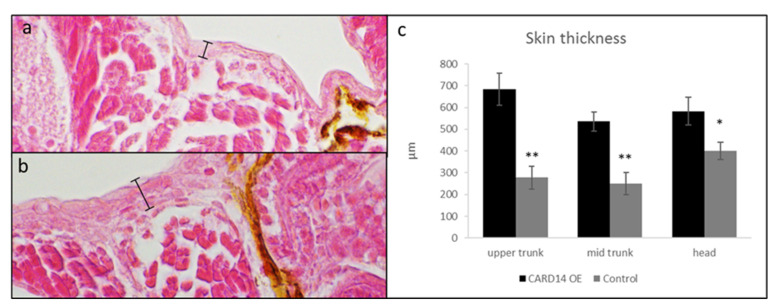
Histopathology at 3 dpf. H&E staining ×40 magnification. Skin of control larvae (**a**) injected with *zfcard14-*RNA containing a stop codon display a thinner epidermis than larvae injected with *zfcard14-*RNA (**b**). Graph (**c**) shows average of 6 measurements at 3 different anatomical sites: head at superior edge of eyes, superior and mid trunk (two-sided *t*-test; * *p* < 0.05, ** *p* < 0.01).

To examine the effect of *CARD14* overexpression on epidermal cellular morphology, we used WISH to determine the expression of the cytokeratin 1-encoding gene. This gene is specifically expressed in the zebrafish developing epidermis and therefore allows for the delineation of keratinocyte morphology. A total of 43 larvae overexpressing *CARD14* and 32 controls were examined, all displaying relatively normal gross development. Keratinocytes of control larvae displayed equal distribution and uniform shape and size, whereas keratinocytes of zf*card14*-overexpressing embryos show an unequal distribution and size. Clusters of cells could be seen in certain areas, and a range of cell size was seen, from small to “giant cells” (Figure 2a–f). To further characterize cutaneous cellular changes, we examined the larvae via scanning electron microscopy. Ten larvae overexpressing either *zfcard14* or *hCARD14* and 10 *gfp* injected controls were examined. Aberrant production of apical microridges was seen. In control embryos, an array of microridges overlie the cell surface that is smooth. In zf*card14*-overexpressing embryos, microridge production is disturbed, and the cell surface displays a mountainous topography, protruding above cell surface, suggestive of hyperkeratosis (Figure 2g–j). Finally, larvae overexpressing zf*card14* displayed thicker skin than control larvae, as revealed by measurements of the skin in larval histological sections (Figure 3).

### 3.4. Over Expression of CARD14 Affects Nfκb and Planar Cell Polarity Signaling Pathways

**Figure 4:**
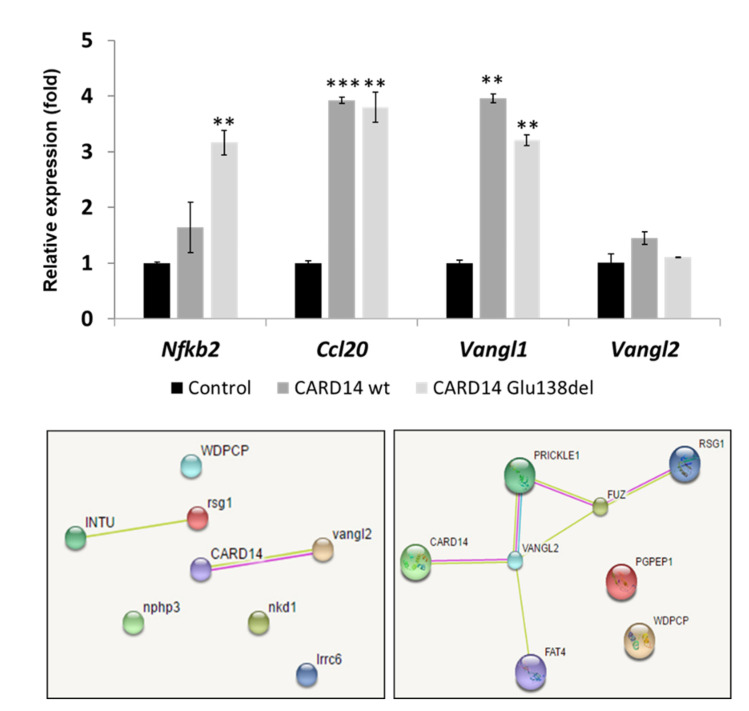
Effect of *CARD14* overexpression on gene expression. Embryos were injected with expression vectors carrying human wild type and PRP-causing *CARD14* mutation (p.Glu138del). Relative expression of *nfkb2*, *ccl20b*, *vangl1* and *vangl2* was ascertained 1 dpf using qPCR (upper panel). Results represent the mean of 3 replicates and are provided as gene expression relative to gene expression in control embryos ± standard error normalized to *ppiab* mRNA levels (two-sided *t*-test; ** *p* < 0.01, *** *p* < 0.001). Protein interaction (STRING, http://string-db.org, accessed on 17 May 2019) analysis of *CARD14* and planar cell polarity proteins in zebrafish (left lower panel) and human (right lower panel) is predictive of a functional interaction between *CARD14* and VANGL2.

Since gain of function mutations in *CARD14* up-regulate Nfκb activity in vitro and in vivo [4,18], we examined the effect of *CARD14* overexpression on this pathway activity in injected embryos. Using, qPCR, we observed a significantly higher expression of *nfkb2*, encoding a light polypeptide gene enhancer in B cells 2 (p49/p100), in injected embryos compared to controls. *ccl20b*, encoding a cytokine induced by Nfκb was up-regulated as well (Figure 4).

The prominent asymmetry of the developing embryos overexpressing CARD14 prompted us to explore the effect of *CARD14* on genes encoding proteins regulating planar cell polarity. Querying the STRING protein interaction prediction program, we found a predicted direct interaction between *CARD14* and the Van Gogh-like protein 2 (VANGL2), a planar cell polarity protein involved in the WNT/PCP pathway (Figure 4). We therefore examined the expression of fish *vangl1*, *vangl2* and *daam1* encoding a protein (Dishevelled Associated Activator Of Morphogenesis 1) expressed downstream to *vangl2*. qPCR showed higher expression of *vangl1* and to a lesser extent *vangl2* in 1 dpf embryos overexpressing *CARD14*, compared to control embryos (Figure 4). Expression of *daam1* did not change. These results suggest CARD14 not only affects the skin, but may alter cephalo-caudal development via interaction with PCP pathway proteins.

## 4. Discussion

The discovery of the role played by CARD14 in familial psoriasis and PRP [4,5] has not only led to the elucidation of the role of this protein in numerous acquired forms of these disorders [5,18,19,20,21,22,23,24,25,26,27,28], but also positions CARD14 as a novel therapeutic and possibly pharmacogenetic target [15,29,30,31]. A practical, robust and rapid model for therapeutic screen is therefore urgently needed. In the present study, we ascertained the zebrafish for its ability to replicate the biological abnormalities seen in humans with gain-of-function mutations in *CARD14*.

Overexpression of *zfcard14* and *hCARD14* in zebrafish revealed marked and reproducible abnormalities in cutaneous morphology including a serrated skin line, spiky pigmentation and a granular surface reflecting the formation of cell aggregates, corresponding to hyperkeratosis. In situ hybridization revealed an uneven size and shape of keratinocytes. Not only were the morphological changes in the developing zebrafish skin reminiscent of the papulosquamous phenotype of psoriasis, gene expression was also consistent with the abnormalities seen in humans with gain-of-function mutations in *CARD14*, as *CARD14* overexpression was clearly associated with up-regulation of the NF-kB pathway in zebrafish as previously seen in humans [4,17].

Interestingly, large scale mutagenesis screens in zebrafish uncovered a mutation initially named *m14* which features keratinocyte hyperplasia resulting in cell aggregates. These findings, reminiscent of our own observations (see above), led investigators to rename this mutation *psoriasis*, because of the phenotypic similarity with the disease [32]. The gene harboring the *psoriasis* mutation has not yet been unequivocally identified. However, it has been mapped at a distance of 8 Mb from the zebrafish *card14* gene [32], suggesting that *card14* may in fact be the gene harboring the *psoriasis* mutation.

Electronic microscopy revealed abnormal topography of keratinocytes’ surface and disturbed microridge production. Microridges, are apical actin protrusions, widely found on vertebrate squamous epithelia [33]. The proposed function of microridges include mucous retention, membrane storage and abrasion resistance [34]. It is largely unknown how microridges are formed, but their formation has been linked to cell polarity pathways [35]. Accordingly, *CARD14* overexpression was associated with up-regulation of NFkB targets as seen in PRP patients [4], but also resulted in altered expression of 2 genes critical for planar cell polarity (PCP), vangl1 and vangl2.

VANGL1 and VANGL2 are evolutionary conserved PCP proteins shown to mediate global cell polarization in different species [36,37]. In zebrafish, *vangl2* has been shown to modulate morphogenetic movements during zebrafish gastrulation through inhibition of the Wnt-β-catenin pathway [38]. In mice, mutations in *Vangl1* and *Vangl2* result in abnormal polarity of cochlear hair cells [39,40]. In humans, mutations in both *VANGL1* and *VANGL2* cause lethal neural tube defects [41,42]. Last but not least, cutaneous effects have been linked to VANGL2 too, as *Vangl2* mutation causes polarization defects of hair growth and direction in mice [43]. VANGL2 was found to interact with proteins of the disheveled-wnt pathway through its PDZ binding domain [44], suggesting a possible interaction with CARD14 which possesses a PDZ domain. Injection of the human *VANGL1* gene was able to partially rescue a pathological phenotype due to down-regulation of vangl2 in zebrafish, indicative of a high degree of functional redundancy of VANGL genes across evolution [45]. Of interest, cell polarity has been suggested to play a role in the pathogenesis of autoinflammatory disorders [46,47], although evidence on the role of polarization in autoimmune diseases is limited. Taken together, CARD14 may possibly play a role in cell polarization and subsequent cephalo-caudal embryonic movements via its interaction with vangl2. The mechanism by which CARD14 mediates polarity and possibly cell migration remains to be studied.

In summary, we have developed a practical model for *CARD14*-overexpression featuring morphological and molecular abnormalities of relevance to *CARD14*-associated human disorders. This model may in the future not only allow for novel therapeutics to be tested in a streamlined fashion, but may also shed new light on the pathogenesis of these conditions.

## Data Availability

The data presented in this study are available on request from the corresponding author.

## Supplementary Materials

The following supporting information can be downloaded at: https://www.mdpi.com/article/10.3390/biomedicines10123192/s1, Table S1: Riboprobes used for WISH; Table S2: Oligonucleotides used for qPCR.
