# Supplementary material for: Cutaneous and Developmental Effects of CARD14 Overexpression in Zebrafish
Source: Biomedicines. 2022 Dec 8;10(12):3192. doi: 10.3390/biomedicines10123192 (PMC9775151; doi:10.3390/biomedicines10123192)
Supplement: Supplementary file 1 [file biomedicines-10-03192-s001.zip › biomedicines-1971985-supplementary.pdf]

biomedicines

Supplementary Materials

Cutaneous and Developmental Effects of *CARD14* Overexpression in Zebrafish

Avital Baniel [1,*], Limor Ziv [2], Zohar Ben-Moshe [3], Ofer Sarig [1], Janan Mohamad [1,4], Alon Peled [1], Gideon Rechavi [2], Yoav Gothilf [3] and Eli Sprecher [1,4]

1 Division of Dermatology, Tel Aviv Sourasky Medical Center, Tel Aviv 64239, Israel
2 Cancer Research Center, Sheba Medical Center, Ramat Gan 52620, Israel
3 Department of Neurobiology, The George S. Wise Faculty of Life Sciences, Tel-Aviv University, Tel Aviv 6997801, Israel
4 Department of Human Molecular Genetics and Biochemistry, Sackler Faculty of Medicine, Tel-Aviv University, Tel Aviv 6997801, Israel
* Correspondence: avital.baniel@gmail.com

Table S1. Riboprobes used for WISH.

krtt1c19e_F	TCAGTGACCTCAACATGGGC
krtt1c19e_R	ATCTCCATCTCCAGCCTGGT
cyt1_F	GCACCCAGATGAGTGGACAA
cyt1_R	CCATCCACCACCTCTTCCAC
krt8_F	ACTGGCAACTGGAATCAAGG
krt8_R	GCACGACAAGAGTGGTGAGA

Table S2. Oligonucleotides used for qPCR.

vangl1_F	TACTGACACTCCTCCTGCTG
vangl1_R	GCAGAGCATCCACCAGTGAT
vangl2_F	CAGCCGCTTCTACAATGTGG
vangl2_R	TGGATTTGGGCAGGTTGAGT
nfkb2_F	AAGCCTGTTGTGTCCAATCC
nfkb2_R	CTTCTCCTCCCAGCACTGAC
daam1b_F	AACTCGTCAAGCAGCAAGTG
daam1b_R	AGAGGACCAGAGACAGGAGG
ppiab_F	TCACACTGAAACACGGAGGCA
ppiab_R	GCTTACCGTCCAGCCAGTTG
