# Supplementary material for: Cutaneous and Developmental Effects of CARD14 Overexpression in Zebrafish
Source: Biomedicines. 2022 Dec 8;10(12):3192. doi: 10.3390/biomedicines10123192 (PMC9775151; doi:10.3390/biomedicines10123192)
Supplement: Supplementary file 1 [file biomedicines-10-03192-s001.zip › Supplementary data.docx]

**Supplementary materials**

**Cutaneous and developmental effects of *CARD14* overexpression in zebrafish**

Avital Baniel^1^, Limor Ziv^2^, Zohar Ben-Moshe Livne^3^, Yoav Gothilf^3^, Ofer Sarig^1^, Janan Mohamad^1,4^, Alon Peled^1^, Gideon Rechavi^2^, Eli Sprecher^1,4^

*^1^Division of Dermatology, Tel Aviv Sourasky Medical Center, Tel Aviv, Israel; ^2^Sheba Medical Center, Ramat Gan, Israel; ^3^The George S. Wise Faculty of Life Sciences, Tel-Aviv University, Ramat Aviv, Israel; ^4^Department of Human Molecular Genetics and Biochemistry, Sackler Faculty of Medicine, Tel-Aviv University, Ramat Aviv, Israel*

**Table S1**

**Riboprobes used for WISH**

| krtt1c19e_F | TCAGTGACCTCAACATGGGC |
| --- | --- |
| krtt1c19e_R | ATCTCCATCTCCAGCCTGGT |
| cyt1_F | GCACCCAGATGAGTGGACAA |
| cyt1_R | CCATCCACCACCTCTTCCAC |
| krt8_F | ACTGGCAACTGGAATCAAGG |
| krt8_R | GCACGACAAGAGTGGTGAGA |

**Table S2**

**Oligonucleotides used for qPCR**

| TACTGACACTCCTCCTGCTG | vangl1_F |
| --- | --- |
| GCAGAGCATCCACCAGTGAT | vangl1_R |
| CAGCCGCTTCTACAATGTGG | vangl2_F |
| TGGATTTGGGCAGGTTGAGT | vangl2_R |
| AAGCCTGTTGTGTCCAATCC | nfkb2_F |
| CTTCTCCTCCCAGCACTGAC | nfkb2_R |
| AACTCGTCAAGCAGCAAGTG | daam1b_F |
| AGAGGACCAGAGACAGGAGG | daam1b_R |
| TCACACTGAAACACGGAGGCA | ppiab_F |
| GCTTACCGTCCAGCCAGTTG | ppiab_R |
